# Multipotent adult progenitor cells prevent functional impairment and improve development in inflammation driven detriment of preterm ovine lungs

**DOI:** 10.1016/j.reth.2024.03.014

**Published:** 2024-03-28

**Authors:** Sophie M.L. Neuen, Daan R.M.G. Ophelders, Helene Widowski, Matthias C. Hütten, Tim Brokken, Charlotte van Gorp, Peter G.J. Nikkels, Carmen A.H. Severens-Rijvers, Mireille M.J.P.E. Sthijns, Clemens A. van Blitterswijk, Freddy J. Troost, Vanessa L.S. LaPointe, Shahab Jolani, Christof Seiler, J. Jane Pillow, Tammo Delhaas, Niki L. Reynaert, Tim G.A.M. Wolfs

**Affiliations:** a Department of Pediatrics, Maastricht University Medical Center, MosaKids Children's Hospital, Maastricht, the Netherlands; b GROW Research Institute for Oncology and Reproduction, Maastricht University, Maastricht, the Netherlands; c Department of BioMedical Engineering, Maastricht University, Maastricht, the Netherlands; d Department of Pathology, University Medical Center Utrecht, Utrecht, the Netherlands; e Department of Pathology, Maastricht University Medical Center, Maastricht, the Netherlands; f Food Innovation and Health, Department of Human Biology, Maastricht University, Venlo, the Netherlands; g NUTRIM Institute of Nutrition and Translational Research in Metabolism, Maastricht University, Maastricht, the Netherlands; h MERLN Institute for Technology-Inspired Regenerative Medicine, Maastricht University, the Netherlands; i Department of Methodology and Statistics, School CAPHRI, Care and Public Health Research Institute, Maastricht University, Maastricht, the Netherlands; j Department of Advanced Computing Sciences, Maastricht University, Maastricht, the Netherlands; k Mathematics Centre Maastricht, Maastricht University, the Netherlands; l School of Human Sciences, University of Western Australia, Perth, WA, Australia; m CARIM School for Cardiovascular Diseases, Maastricht University, Maastricht, the Netherlands; n Department of Respiratory Medicine, Maastricht University, Maastricht, the Netherlands

**Keywords:** Prenatal inflammation, Postnatal ventilation, Preterm birth, Bronchopulmonary dysplasia, Stem cell therapy

## Abstract

**Background:**

Perinatal inflammation increases the risk for bronchopulmonary dysplasia in preterm neonates, but the underlying pathophysiological mechanisms remain largely unknown. Given their anti-inflammatory and regenerative capacity, multipotent adult progenitor cells (MAPC) are a promising cell-based therapy to prevent and/or treat the negative pulmonary consequences of perinatal inflammation in the preterm neonate. Therefore, the pathophysiology underlying adverse preterm lung outcomes following perinatal inflammation and pulmonary benefits of MAPC treatment at the interface of prenatal inflammatory and postnatal ventilation exposures were elucidated.

**Methods:**

Instrumented ovine fetuses were exposed to intra-amniotic lipopolysaccharide (LPS 5 mg) at 125 days gestation to induce adverse systemic and peripheral organ outcomes. MAPC (10 × 10^6^ cells) or saline were administered intravenously two days post LPS exposure. Fetuses were delivered preterm five days post MAPC treatment and either killed humanely immediately or mechanically ventilated for 72 h.

**Results:**

Antenatal LPS exposure resulted in inflammation and decreased alveolar maturation in the preterm lung. Additionally, LPS-exposed ventilated lambs showed continued pulmonary inflammation and cell junction loss accompanied by pulmonary edema, ultimately resulting in higher oxygen demand. MAPC therapy modulated lung inflammation, prevented loss of epithelial and endothelial barriers and improved lung maturation *in utero*. These MAPC-driven improvements remained evident postnatally, and prevented concomitant pulmonary edema and functional loss.

**Conclusion:**

In conclusion, prenatal inflammation sensitizes the underdeveloped preterm lung to subsequent postnatal inflammation, resulting in injury, disturbed development and functional impairment. MAPC therapy partially prevents these changes and is therefore a promising approach for preterm infants to prevent adverse pulmonary outcomes.

## Highlights

• Prenatal inflammation sensitizes the immature lung to postnatal inflammatory stress.
• Perinatal lung inflammation hinders development and function of the immature lung.
• MAPC therapy regenerates structure of the immature lung following inflammation.
• MAPC therapy restores function of the inflamed preterm lung.
• MAPC therapy induces maturation in the developing alveoli.

## Abbreviations

AAG: alveolar-arterial gradient
AEC: alveolar epithelial cells
AS: alveolar space
AW: alveolar wall
BAL: bronchioalveolar lavage
BALf: bronchioalveolar lavage fluid
BPD: bronchopulmonary dysplasia
CD: cluster of differentiation
FIO2: fraction of inspiratory oxygen
dGA: days of gestational age
GAPDH: glyceraldehyde 3-phosphate dehydrogenase
i.a.: intra-amniotic
IBA-1: ionized calcium-binding adapter molecule 1
IL: interleukin
IQR: interquartile range
i.v.: intra-venous
LPS: lipopolysaccharide
LLL: left lower lobe
LUL: left upper lobe
MAPC: multipotent adult progenitor cells
MSC: mesenchymal stromal cells
MV: mechanical ventilation
ovRSP15: ovine ribosomal protein S15
PaCO2: partial pressure of carbon dioxide
PaO2: partial pressure of oxygen
Patm: atmospheric pressure
PC: principal component
PCA: principal component analysis
PEEP: positive end-expiratory pressure
PH2O: water pressure
PIP: positive inspiratory pressure
RAC: radial alveolar count
RLL: right lower lobe
RUL: right upper lobe
RQ: respiratory quotient
SOX: SRY-related HMG-box
TTF-1: thyroid transcription factor 1
YWHAZ: Human 14-3-3 protein zeta/delta

## 1 Background

Bronchopulmonary dysplasia (BPD) is a chronic inflammatory lung disease in preterm infants, leading to lasting pulmonary impairment [1]. Its etiology is multifactorial, and both prenatal- and postnatal mediators contribute to adverse outcomes. Preterm infants born from chorioamnionitis-complicated pregnancy have an increased risk for BPD [1,2]. Preterm newborns frequently need respiratory support, but when applied for an extended period, it exacerbates pulmonary inflammation. Importantly, the combination of chorioamnionitis with postnatal inflammatory triggers such as mechanical ventilation (MV) are synergistic, and increase the risk for BPD [2,3]. The pathophysiology of BPD involves a complex interplay between factors, including pulmonary inflammation interrelated with oxidative stress and endothelial and epithelial dysfunction, resulting in the hallmarks of BPD, namely developmental arrest and injury of the lung [4]. However, the molecular events leading to damage and arrested development of the immature lung during perinatal inflammation are incompletely understood, hampering the development of novel therapeutic strategies.

Currently, neonatal respiratory therapies do not prevent long-term pulmonary impairment after perinatal inflammation. In addition, immunomodulatory therapies such as postnatal glucocorticoids have short-term benefit, but significant concerns remain on their side effects [4,5]. Given the central roles of inflammation, injury and arrested development in the pathogenesis of adverse outcomes of the preterm lung, an efficient treatment should prevent or reduce inflammation, and induce regenerative pathways to counteract lung injury and restore development.

Bone marrow derived mesenchymal stromal cells (MSC) emerged recently as a promising therapeutic strategy for BPD due to their anti-inflammatory and regenerative properties, and their off-the-shelf availability [6,7]. The efficacy along with the safety of MSC administration are described in postnatal rodent models in which BPD hallmarks were induced by exposing pups to hyperoxia. In these studies, MSC exhibited anti-inflammatory properties [8,9], and enhanced alveolar development [10,11]. These preclinical results formed the basis for current Phase I and Phase II clinical trials [12,13]. These first dose-escalation clinical studies primarily focus on the optimal dose, administration route and safety of MSC in preterm neonates either at risk for BPD or who have had BPD diagnosed [13]. Therefore, multiple essential questions remain unanswered, including the pharmacological regime and working mechanisms of MSC [12].

Given the multifactorial etiology of BPD, we developed a preclinical ovine model, which closely recapitulates the clinical setting of prematurity-associated adverse lung outcomes. This preterm ovine model was used to study the pathogenesis of adverse lung outcomes both during pregnancy and directly after birth. Moreover, physiological and anatomical similarities exist between sheep and human lung development, which are essential for an efficient translation of preclinical findings [14,15]. To this end, perinatal inflammatory stress was induced by prenatal intrauterine inflammation and postnatal mechanical ventilation following preterm birth mimicking the multifactorial pathogenesis of postnatal adverse lung outcomes, such as BPD. Firstly, we aimed to examine the cascade of antenatal and postnatal processes that contribute to adverse outcomes of the immature lung. Secondly, we aimed to study the effect of systemic multipotent adult progenitor cell (MAPC) administration on pulmonary function and development when given at the interface of two consecutive pro-inflammatory insults, meaning after prenatal lipopolysaccharide (LPS) exposure but before postnatal MV.

## 2 Methods

### 2.1 Study approval

Animal experiments were approved by the Dutch Central Authority for Scientific Procedures on Animals (AVD107002016784) and the Animal Welfare Body of Maastricht University (PV2016-016).

### 2.2 Prenatal interventions

**Fig. 1:**
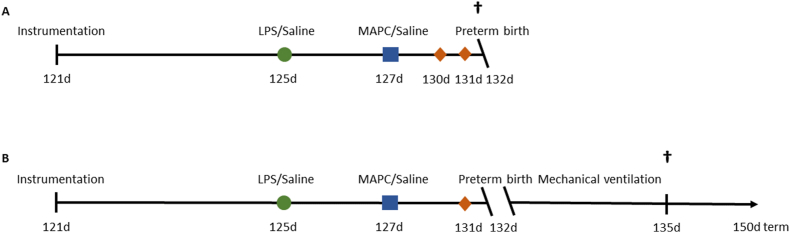
Study design of pre-clinical ovine study for perinatal stress and prenatal MAPC treatment. After the instrumentation at 121 dGA and a recovery period of 4 d, either a bolus of LPS or saline as control (green) was administered i.a. At 127 dGA, the fetus received an i.v. bolus of MAPC (blue) or saline as control. Betamethasone (orange), was administered 24 h before birth and the prenatal cohort received an additional dose 48 h prior to birth (A). At 132 dGA, the premature lamb was delivered via Cesarean section and lambs of the prenatal cohort were humanely killed (A). Lambs of the postnatal cohort were mechanically ventilated after birth (B). After 72 h of MV, the animals of the postnatal cohort were humanely killed (postnatal assessment cohort; B). dGA = days of gestational age, LPS = lipopolysaccharides, MAPC = multipotent adult progenitor cells, MV = mechanical ventilation, i.a. = intra-amniotic, i.v. = intra-venous.

Forty-eight singletons of time-mated Texel ewes were randomized into the prenatal- or postnatal sub-study (Fig. 1). Surgical instrumentation of fetuses (121 days of gestational age (dGA)), i.a. LPS injection (125 dGA), MAPC preparation and administration (127 dGA) and betamethasone (11.4 mg i.m., Celestone®; Schering Plough Labo NV, Heist-op-den-Berg, BE) administration 24 h before birth and additionally at 48 h before birth for the prenatal cohort were performed as previously reported [16]. At 132 dGA (term 147 dGA), which is comparable to 33 weeks of human gestation and lung development is in the saccular phase [15], fetuses were surgically delivered preterm, and either killed humanely immediately with pentobarbital (200 mg/kg i.v., AST Farma B.V., Oudewater, NL) or mechanically ventilated for 72 h prior to humane killing. All ewes were killed humanely with pentobarbital (200 mg/kg i.v. AST Farma B.V., Oudewater, NL).

### 2.3 Postnatal interventions

Twenty-two pregnant ewes were randomized to the postnatal study (n = 4 to 6/group): a smaller Sham-MAPC group of n = 4 was used in the postnatal study to exclude MAPC-induced alterations in the lungs in a control setting, since previous clinical and experimental studies extensively showed the safety of MAPC under control conditions [7,16]. Fetuses of the postnatal cohort received identical prenatal treatments to the fetuses of the prenatal cohort, according to their group allocation. At 132 dGA, fetuses were surgically delivered, which was followed by oral intubation with an endotracheal tube, umbilical cord clamping and the lambs were weighted. Lambs were transitioned to an infant radiator bed (IW930 Series, Cosy-Cots™ Infant Warmer, Fisher&Paykel, Auckland, NZ), where they were dried and placed in a sling [17]. The lamb was immediately connected to an infant ventilation device (Fabian HFO®; Acutronic, Hirzel, CH) [18]. Lambs were ventilated according to a volume-controlled strategy, with the following initial ventilator settings: synchronized intermittent mandatory ventilation (SIMV), F_I_O_2_ of 40%, breathing frequency of 50 breaths/min, positive end-expiratory pressure of 8 mmHg, maximal positive inspiratory pressure of 30 mmHg, and an inspiratory volume of 6 mL/kg. The lamb received surfactant at 15 min via an endotracheal administration (200 mg/kg BW, Poractant alpha Curosurf®, Chiesi Pharmaceuticals, Parma, IT) [18], antibiotics at 30 min and blood transfusion at 60 min after the start of ventilation. To minimize the risk of an infant respiratory distress syndrome, which is associated with the gestational age of 132 d and the concomitant immaturity of the lungs, surfactant was given prophylactically. Lambs received antibiotics to prevent infections that could arise from instrumentation and intubation, as the immunity of lambs develops only with the intake of colostrum. During the ventilation period, blood was drawn several times and therefore lambs received a maternal blood transfusion to compensate the blood loss. To reduce confounders all lambs received surfactant, antibiotics and blood transfusion. After the first hour of ventilation, the ventilation settings were adjusted based on blood gas analysis (iStat device; Point of Care, Inc., Abbot Park, IL) and the respiratory needs of the animals. Hereby a PaO_2_ range of 60–90 mmHg and a PaCO_2_ range of 45–70 mmHg were used as target values [18]. Thereafter, blood gas analysis was performed every hour after a change in ventilation settings occurred or every 3 h when the ventilation parameters were not adapted.

Animals were ventilated continuously for 72 h, whereby ventilation data were recorded every hour throughout the experiment, as well as body temperature, heart frequency and arterial blood pressure. During these 72 h, lambs received parental feeding intravenously via the femoral vein, consisting of a mixture of 20% glucose (Baxter BV, Utrecht, NL), 10% primene (Baxter B.V., Utrecht, NL), 10% calcium gluconate (B. Braun, Melsungen, DE) and 15% magnesium sulfate (Kalcex, Riga, LV), and were additional fed with maternal colostrum and/or colostrum replacement (Colstart Plus, Holland animal care, Enter, NL). Lambs went through a natural cycle of feeding and resting without the need of sedation. To counteract the discomfort of the intubation, animals received buprenorphine (0.1 mg/mL, Ecuphar, Oostkamp, BE), every 6 h. 72 h after delivery, lambs were humanely killed with pentobarbital (200 mg/kg, i.v.).

The ventilation data and blood gas results obtained over 72 h were used to calculate the alveolar–arterial (A–a) gradient according to the following equation [19]:

$$A-a\;gradient=\left[\left(F_{I}O_{2}\;x\;\left\{Patm-PH_{2}0\right\}\right)-\left(\frac{PaCO_{2}}{RQ}\right)\right]-PaO_{2}$$

Hereby, assuming that the atmospheric pressure (Patm) is 760 mmHg, water pressure (PH_2_O) is 47 mmHg and the respiratory quotient (RQ) is 0.8. The PaO_2_ and the PaCO_2_ were acquired by means of arterial blood gas analyses. The F_I_O_2_ was a manually changeable parameter on the ventilator device, which was adjusted when PaO_2_ values exceeded the predefined range of 60–90 mmHg, and was additionally presented for the course of 72 h in percentage.

### 2.4 Post-mortem tissue sampling and analyses

The entire right lung was inflated and fixated at 30 cm H_2_O in 4% paraformaldehyde for 24 h. The left upper lobe (LUL) was snap-frozen in liquid nitrogen and the left lower lobe (LLL) was used for bronchioalveolar lavage (BAL) with 0.9% sodium chloride. BAL fluid was centrifuged at 500×*g* for 10 min at 4 °C and the supernatant was stored at −80 °C.

Circulatory IL-6 concentrations were determined in plasma and IL-8 was measured in BAL fluid (BALf) with an in-house ELISA [16,20]. Glutathione (GSH) and glutathione disulfide (GSSG) concentrations were measured in BALf using the enzymatic recycling method [21].

Paraffin-embedded 4 μm lung sections of the right upper lobe (RUL) and right lower lobe (RLL) were stained with hematoxylin and eosin to visualize histological structures. Immunohistochemistry was performed for immune cells by CD45, macrophages by IBA-1 and alveolar epithelial cells type 2 (AEC2) by TTF-1 [22]. Antibodies are listed in Supplementary Table 2.

### 2.5 Morphological analyses

**Supplementary Fig. 1:**
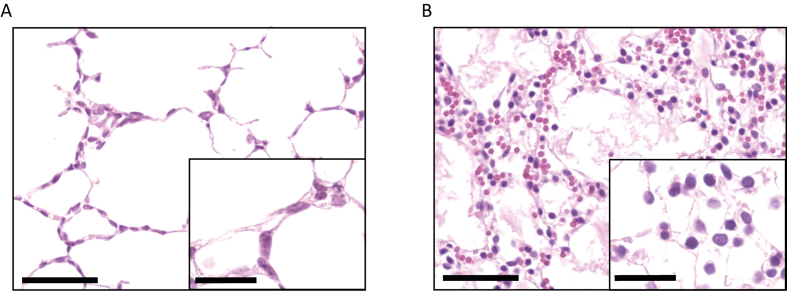
Edema scoring. Representative histological images are shown for edema score 0 (no edema) (A) and score 3 (alveolar congestion/flooding of alveoli) (B). Image magnification is 200× and for insets 400×. Scale bars: 50 μm; scale bars in insets: 20 μm.

Multi-layered scans with a magnification of 200× were acquired for morphological analysis using a Ventana iScan slide scanner (Ventana Medical Systems, Oro Valley, AZ, USA). Regions of interest were restricted to the alveolar compartment. All analyses were performed by two blinded observers with the open-source digital pathology software QuPath (v0.3.2) [23]. Alveolarization and edema were assessed using radial alveolar counts (RAC) [24] and according to Slavin et al. [25] (Supplementary Table 3), respectively. Representative histological images for the edema scoring can be found in Supplementary Figure 1.

CD45- and TTF-1-positive cell count was corrected for tissue surface area. IBA-1 immunoreactivity is presented as the positive percentage of tissue area per high-power field.

### 2.6 RNA extraction and quantitative real-time RT-qPCR

Snap frozen lung tissue of the left upper lobe was homogenized for RNA isolation to analyze the mRNA sequences summarized in Supplementary Table 1.

RNA extraction was performed with a Qiagen RNeasy Mini Kit (74104, Qiagen, Hilden, DE). cDNA was synthesized with the SensiFastTM cDNA Synthesis Kit (BIO-65054, Meridian Bioscience, Luckenwalde, DE). Real-time qPCR was performed, with the SensiMixTM SYBR Hi-Rox Kit (QT605, Meridian Bioscience, Luckenwalde, DE) and LightCycle 480 Instrument (Roche Applied Science, Basel, CH). The Geomean of the housekeeping genes was applied to normalize the results. Data are presented as fold changes in mRNA levels compared to controls (calculated from N0 values based on LinRegPCR).

### 2.7 Western blot

Crushed frozen tissue was homogenized in RIPA buffer (R0278, Sigma-Aldrich, St. Louis, MO, USA), supplemented with 0.1% protease (cOmplete, Roche, Basel, CH) and phosphatase inhibitor (PhosStop, Roche, Basel, CH), and equalized to a protein content of 1.8 μg/μl. Following denaturation and reduction, homogenized lung samples (15 μl) were loaded onto 4–12% Bis-Tris Criterion gels (3450125, Bio-Rad, Veenendaal, NL). After the transfer of proteins to nitrocellulose (162-0115, Bio-Rad, Veenendaal, NL), membranes were blocked with 5% milk (ELK, Friesland Campina, Amersfoort, NL). Blots were incubated overnight with the primary antibodies (details described in Supplementary Table 2) at 4 °C, followed by incubation with the secondary antibody (details described in Supplementary Table 2). Protein bands were visualized with Pico Chemiluminescent Substrate kit (34580, Thermo Fisher, Breda, NL) and intensities quantified using ImageQuant TL (v8.1.0.0). Protein contents of β-actin were used to normalize the data and presented as fold changes compared to controls.

### 2.8 Statistical analysis

Kruskal-Wallis test with Dunn's multiple comparison was performed on end-point data (v6.0, GraphPad software Inc., La Jolla, CA, USA). Ventilation indices were analyzed using linear mixed-effects model (IBM SPSS Statistics Software, v28.0.1, IBM. Armonk, NY, USA), based on the natural logarithm of the MV measurements. Hereby, LPS exposure and MAPC treatment were fixed effects and MV time points were regarded as a continuous measurements over 72 h. Differences were regarded as statistically significant at *p* < 0.05. Considering the relatively small number of animals per group *p*-values between 0.05 and 0.1 were interpreted as biologically relevant and exact *p*-values are reported [20,26]. Data clustering was investigated with Principal Component Analysis (PCA) using R (v.4.2.2).

## 3 Results

### 3.1 Animal characteristics

Group sizes and animal characteristics are displayed in Table 1. No significant differences were found in sex, birth weight and post-mortem lung-to-bodyweight ratio. Prenatal LPS exposure significantly elevated IL-6 concentrations 24 h post-LPS exposure compared to control groups (Prenatal study: LPS-Saline *p* = 0.023; LPS-MAPC *p* = 0.045. Postnatal study: LPS-Saline-MV *p* = 0.02; LPS-MAPC-MV *p* = 0.002).

**Table 1:** Animal characteristics.

Prenatal cohort (*n* = 21)	Sham-Saline	Sham-MAPC	LPS-Saline	LPS-MAPC
*n* (male %)	5 (40)	5 (60)	6 (33.33)	5 (3.33)
Birthweight (kg)	3.1 (1.8, 3.6)	3.0 (2.5, 3.4)	2.8 (2.5, 3.1)	3.5 (3.1, 3.7)
Lungwt/bodywt (g/kg)	24.48 (20.74, 30.59)	29.15 (25.29, 34.46)	26.55 (22.08, 30.29)	24.89 (22.95, 29.72)
IL-6 (pg/mL)^a^	1.0 (1.0, 78.74)	1.0 (1.0, 47.72)	331.2 (274.5, 697.1)∗	335.0 (205.1, 882.6)∗
**Postnatal cohort (*n* = 22)**	**Sham-Saline-MV**	**Sham-MAPC-MV**	**LPS-Saline-MV**	**LPS-MAPC-MV**
*n* (male %)	6 (33.3)	4 (75)	6 (66.67)	6 (50)
Birthweight (kg)	2.4 (2.2, 3.1)	3.3 (2.8, 3.7)	3.0 (2.4, 3.2)	3.0 (2.6, 3.4)
Lungwt/bodywt (g/kg)	36.21 (31.74, 45.21)	35.81 (28.11, 46.40)	40.09 (32.93, 45.31)	35.02 (32.61, 41.1)
IL-6 (pg/mL)^a^	1.0 (1.0, 96.53)	1.0 (1.0, 1.0)	180.1 (106.0, 375.80)∗	544.8 (180.5, 740.8)∗∗

Data are presented as median with IQR. Statistical significance was determined with a Kruskal-Wallis test followed by Dunn's multiple comparison and depicted as ∗*p* < 0.05 and ∗∗*p* < 0.01 compared to Sham-Saline.

IQR = interquartile range, MAPC = multipotent adult progenitor cells, MV = mechanical ventilation.

a IL-6 concentrations measured 24 h post i.a. LPS/Saline.

### 3.2 Oxygenation

The F_I_O_2_ was recorded and the alveolar–arterial (A–a) gradient was calculated as functional readouts for oxygenation over the 72 h postnatal ventilation. MV was based on PaO_2_ range of 60–90 mmHg and SpO_2_ targets of ≥95%.

**Fig. 2:**
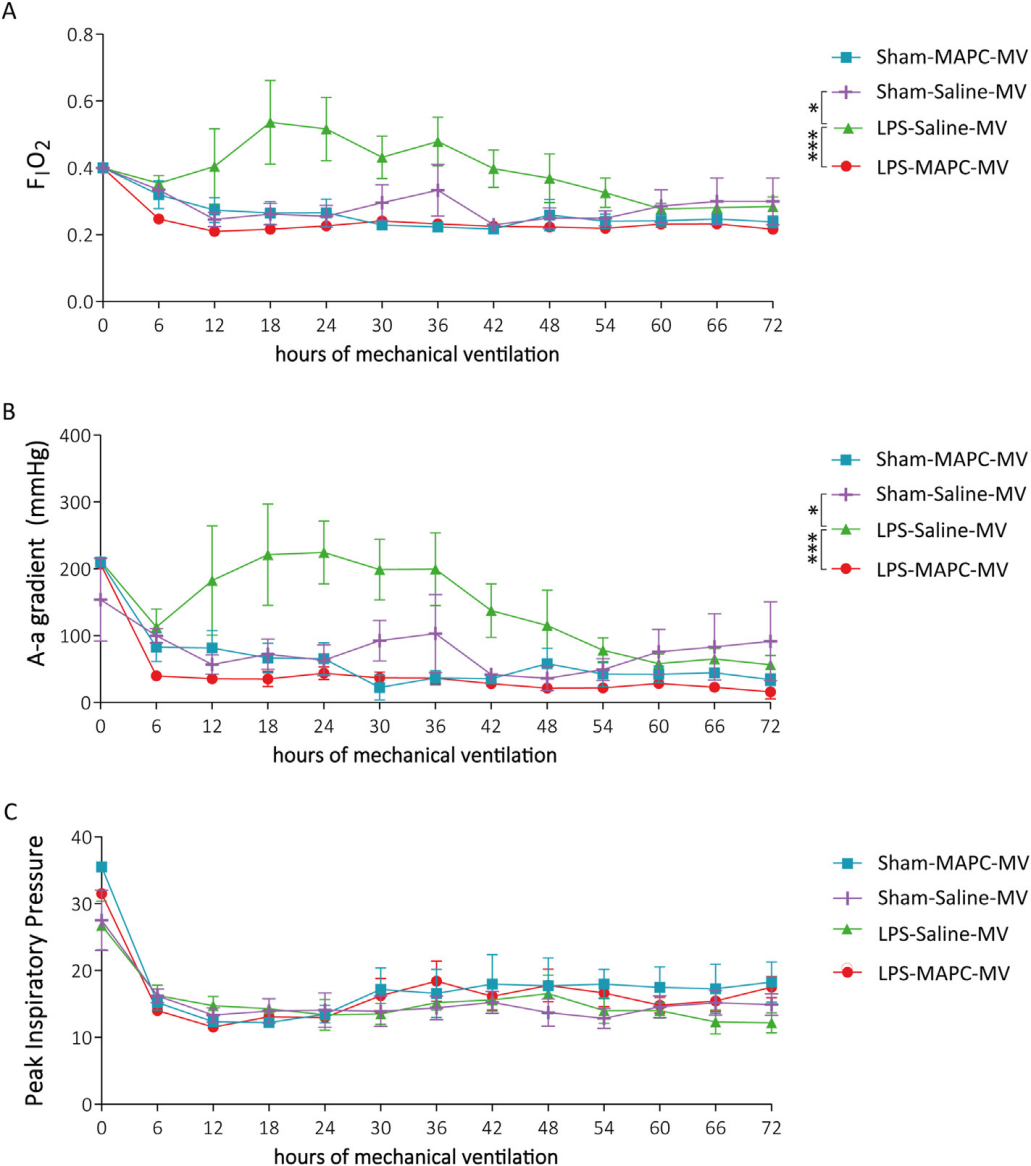
Perinatal inflammation results in elevated oxygen requirement, which is prevented by MAPC treatment. Prenatal LPS exposure with subsequent postnatal MV led to increased F_I_O_2_ (A), as well as an increased A-a gradient (B), which was prevented by MAPC treatment (A & B). PIP did not differ between groups (C). Data are presented as mean ± standard deviation. Statistical significance was determined with linear mixed effects models analysis and MV time points were regarded as continuous measurements over 72 h. Significance is indicated by ∗*p* < 0.05, ∗∗∗*p* < 0.001. A–a gradient = arterial–alveolar gradient, F_I_O_2_ = fraction of inspired oxygen, MAPC = multipotent adult progenitor cells, MV = mechanical ventilation, PIP = peak inspiratory pressure.

In unexposed animals, the F_I_O_2_ decreased after the first hour of MV and remained stable throughout the whole course at 0.21–0.25 (Fig. 2A). Lambs exposed to LPS prior to MV had significantly elevated F_I_O_2_ requirements (0.4–0.6) from 12 h until 60 h compared to controls (*p* = 0.022 Sham-Saline-MV vs LPS-Saline-MV; Fig. 2A). In contrast, MAPC treatment resulted in complete prevention of the higher oxygen demand created by prenatal LPS exposure (*p* = 0.001 LPS-Saline-MV vs LPS-MAPC-MV; Fig. 2A), with oxygen requirements comparable to unexposed animals.

Consistent with the higher F_I_O_2_ levels, the A–a gradient of LPS-exposed animals was higher between 12 h and 48 h of MV compared to non-LPS-exposed controls (*p* = 0.031 Sham-Saline-MV vs LPS-Saline-MV; Fig. 2B). MAPC treatment following LPS exposure again completely prevented this elevation and the A–a gradient remained comparable to baseline (*p* < 0.001 LPS-Saline-MV vs LPS-MAPC-MV; Fig. 2B).

In peak inspiratory pressures (PIP) no significant differences between groups were found (Fig. 2C).

### 3.3 Immune response

Inflammation is one of the key drivers behind the etiology of BPD, which prompted us to examine the pulmonary immune response. Differences in the location of both CD45^+^ immune cells and IBA-1+ macrophages were assessed taking the pro- and anti-inflammatory properties of these cells into account.

**Fig. 3:**
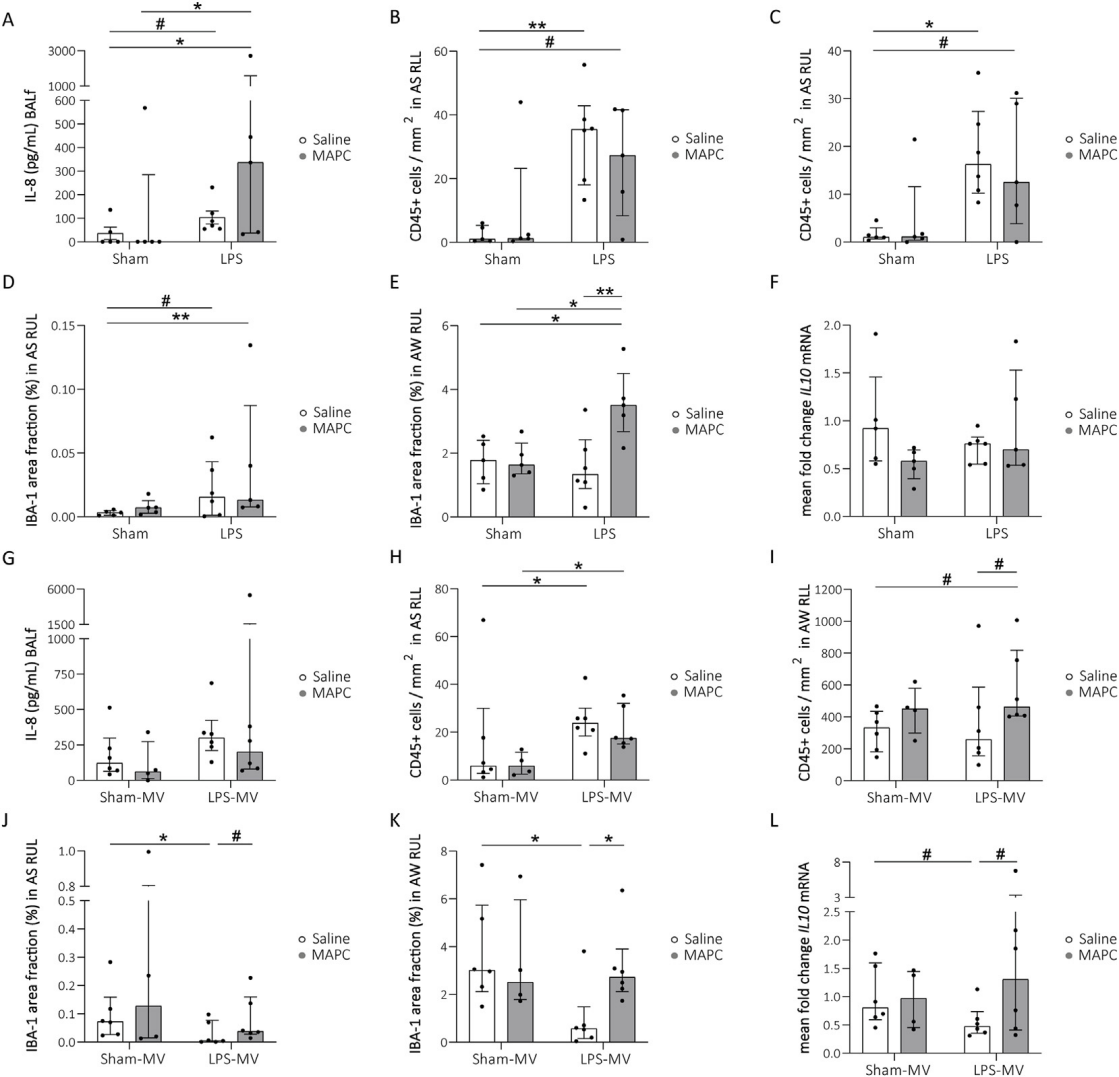
Prenatal LPS exposure increases the pulmonary pro-inflammatory immune response at birth and MAPC modulate the immune response to LPS and/or MV in preterm ovine lungs. Prenatal LPS exposure led to increased protein levels of the pro-inflammatory IL-8 in BALf (A). LPS exposure also increased the numbers of CD45^+^ immune cells in the AS of the RLL (B) and of the RUL (C), as well as the IBA-1 immunoreactivity in the AS of the RUL (D). In the AW of the RUL, more IBA-1 immunoreactivity was present in MAPC-treated LPS-exposed animals of the prenatal cohort (E). *IL10* mRNA expression levels were unchanged by both LPS exposure and MAPC treatment (F). IL-8 levels did not differ between ventilated groups (G). However, prenatal LPS exposure in combination with MV resulted in higher numbers CD45^+^ immune cells residing in the AS of the RLL (H). MAPC therapy of LPS-exposed animals resulted in a biological relevant increase in the number of CD45^+^ immune cells in the AW following 72 h of MV (I). LPS exposure combined with postnatal MV, resulted in less IBA-1immunoreactivity in both the AS (J) and the AW (K), which was prevented by MAPC treatment. A biological relevant decrease in *IL10* mRNA levels was seen in LPS-exposed animals, which was biologically relevant obviated by MAPC therapy (L). Data are presented as median with IQR. *IL10* mRNA levels are displayed as relative fold changes over the Sham-Saline group and Sham-Saline-MV group, respectively. Statistical significance was determined with a Kruskal-Wallis test followed by Dunn's multiple comparison and depicted as #*p* < 0.1, ∗*p* < 0.05. BALf = bronchioalveolar lavage fluid, AS = alveolar space, AW = alveolar wall, IQR = interquartile range, MAPC = multipotent adult progenitor cells, MV = mechanical ventilation, RLL = right lower lobe, RUL = right upper lobe.

Following elevated systemic IL-6 levels (Table 1) at 24 h post-LPS exposure, pulmonary inflammation was present at birth with increased IL-8 levels in BALf compared to non-exposed controls (IL-8: *p* = 0.089 Sham-Saline vs LPS-Saline, *p* = 0.043 Sham-Saline vs LPS-MAPC; Fig. 3A). Furthermore, CD45^+^ cell counts were elevated in the alveolar space (AS) (right lower lobe (RLL): *p* = 0.01 Sham-Saline vs LPS-Saline, *p* = 0.053 Sham-Saline vs LPS-MAPC; Fig. 3B; right upper lobe (RUL): *p* = 0.014 Sham-Saline vs LPS-Saline, *p* = 0.092 Sham-Saline vs LPS-MAPC; Fig. 3C) and IBA-1 immunoreactivity, indicative for macrophages, increased in the AS of the RUL after LPS-exposure (*p* = 0.073 Sham-Saline vs LPS-Saline, *p* = 0.01 Sham-Saline vs LPS-MAPC; Fig. 3D).

Since pulmonary interstitial macrophage populations have crucial immunoregulatory properties [27,28], IBA-1 immunoreactivity and the number of CD45^+^ cells were also examined in the alveolar wall (AW). Although LPS-exposure did not affect assessed immune cells in the AW at birth (Supplementary Fig. 1A and B), MAPC treatment in LPS-exposed lambs significantly increased IBA-1 immunoreactivity in AW of the RUL at birth (*p* = 0.024 LPS-MAPC vs Sham-Saline; *p* = 0.037 LPS-MAPC vs Sham-MAPC; Fig. 3E).

mRNA expression of the anti-inflammatory cytokine *IL10* was measured to further unravel the immunomodulatory effect of MAPC. No changes in *IL10* mRNA expression were observed at birth (Fig. 3F).

**Fig. 4:**
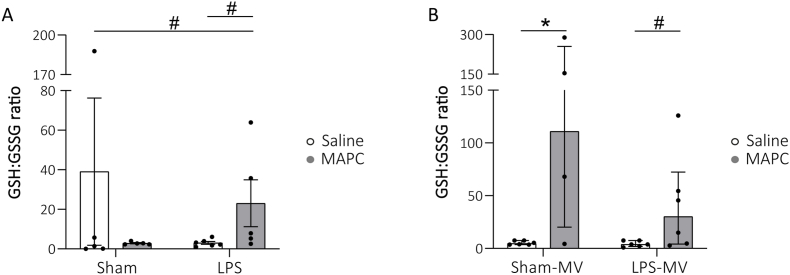
MAPC modify the redox status in the pulmonary epithelial lining fluid. MAPC therapy following i.a. LPS exposure resulted in a biological relevant increase of the GSH:GSSG ratio compared to untreated animals (A). Also during postnatal assessment, the increase in GSH:GSSG ratio was observed and interestingly, unexposed MAPC-treated animals displayed higher GSH:GSSG levels compared to controls (B). GSH and GSSG concentrations were determined in BALf. Data are presented as median with IQR. Statistical significance was determined with a Kruskal-Wallis test followed by Dunn's multiple comparison and depicted as #*p* < 0.1, ∗*p* < 0.05. BALf = bronchioalveolar lavage fluid, i.a. = intra-amniotic, IQR = interquartile range, MAPC = multipotent adult progenitor cells, MV = mechanical ventilation.

Next we examined, whether the increase in pro-inflammatory markers following LPS exposure and the counteracting anti-inflammatory effects of MAPC continued three days post MV. No difference in IL-8 levels three days post-MV were observed (Fig. 3G). However, a persistent increase of CD45^+^ cells was found in AS of the RLL of LPS-exposed animals compared to unexposed animals (*p* = 0.045 Sham-Saline-MV vs LPS-Saline-MV, *p* = 0.035 Sham-MAPC-MV vs LPS-MAPC-MV; Fig. 3H). MAPC therapy post-LPS exposure resulted in a biologically relevant elevation of the numbers of CD45^+^ cells in the AW of the RLL (*p* = 0.068 Sham-Saline-MV vs LPS-MAPC-MV, *p* = 0.083 LPS-Saline-MV vs LPS-MAPC-MV; Fig. 4I).

IBA-1 immunoreactivity decreased in both the AS and AW of the RUL in ventilated LPS-exposed animals compared to unexposed animals (AS: *p* = 0.037 Sham-Saline-MV vs LPS-Saline-MV; AW: *p* = 0.015 Sham-Saline-MV vs LPS-Saline-MV; Fig. 3J and K). MAPC therapy prevented this decrease in IBA-1 immunoreactivity in the AW (*p* = 0.037 LPS-Saline-MV vs LPS-MAPC, Fig. 3K) and a similar biologically relevant prevention was observed in the AS (*p* = 0.075 LPS-Saline-MV vs LPS-MAPC-MV; Fig. 3J). In the RLL, no changes in AW IBA-1 immunoreactivity were seen (Supplementary Fig. 1C and D).

Prior LPS exposure resulted in a biological relevant decrease *IL1*0 mRNA expression in ventilated animals (*p* = 0.083 Sham-Saline-MV vs LPS-Saline-MV). MAPC treatment lead to a biologically relevant prevention of the LPS-induced decrease in *IL1*0 mRNA levels (*p* = 0.062 LPS-Saline-MV vs LPS-MAPC-MV; Fig. 4B).

### 3.4 Redox status

In this study we investigated oxidative stress in the lungs, which is involved in the development of BPD, based on the redox status of glutathione. In lambs of the prenatal cohort, MAPC administration following LPS exposure resulted in a biological relevant increase in the GSH:GSSG ratio compared to untreated animals (*p* = 0.059 Sham-Saline vs LPS-MAPC, *p* = 0.068 LPS-Saline vs LPS-MAPC; Fig. 4A). After three days of MV, GSH:GSSG ratios were higher in MAPC-treated animals compared to untreated animals (*p* = 0.033 Sham-Saline-MV vs Sham-MAPC-MV, *p* = 0.083 LPS-Saline-MV vs LPS-MAPC-MV; Fig. 4B), signifying a higher glutathione redox potential.

### 3.5 Edema and cell junctions

**Fig. 5:**
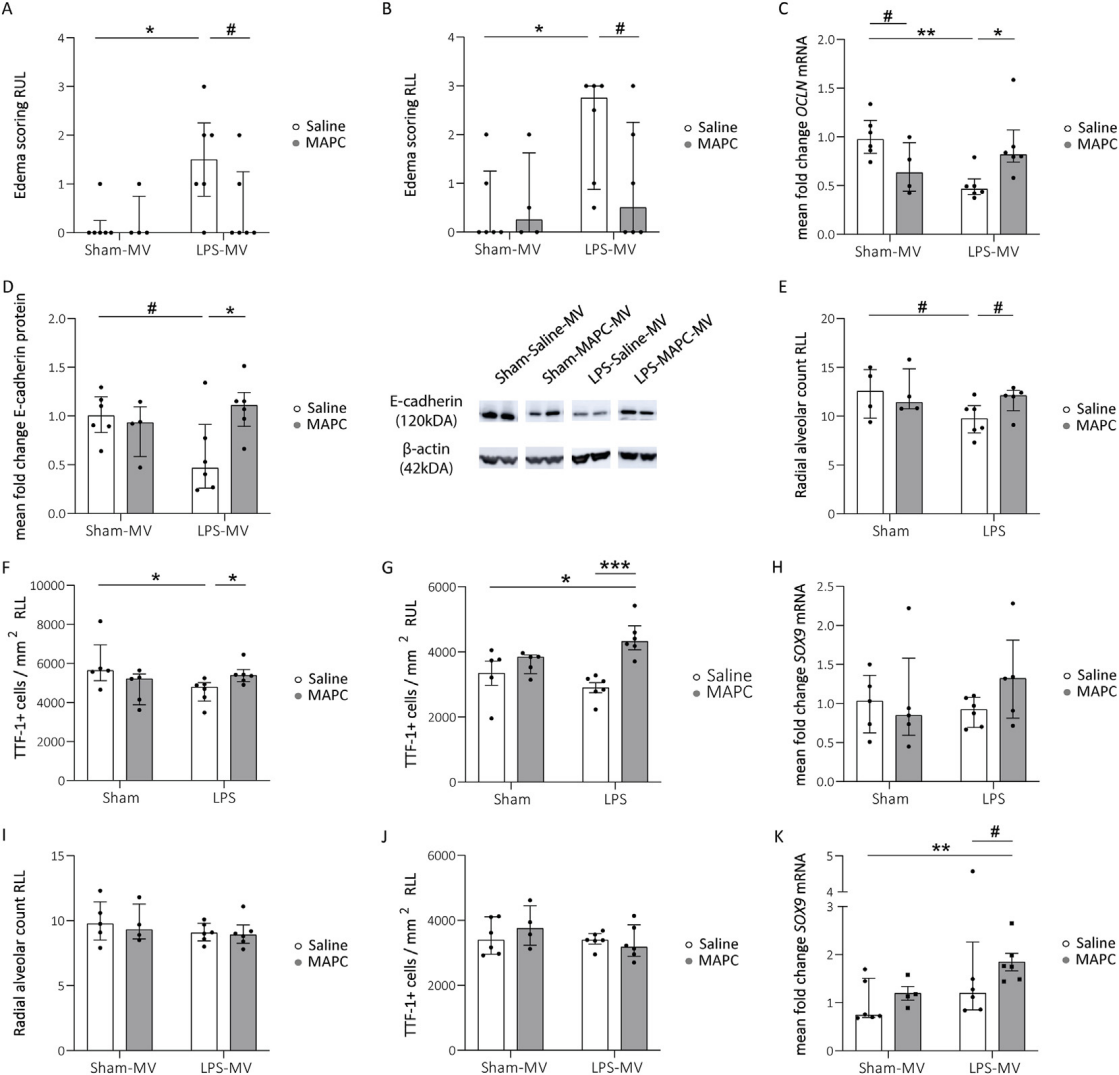
Edema accompanied by cell-junctional loss following perinatal inflammation is counteracted by MAPC treatment and MAPC therapy enhances distal alveolar development. Edema formation was observed in both the RUL (A) and the RLL (B) of preterm ovine lungs, which were exposed to prenatal LPS and postnatal MV. Simultaneously, in MAPC-treated animals, the excess of fluid was reduced (A & B). OCLN expression was disturbed following LPS exposure and normalized after MAPC treatment (C). Similarly, LPS-exposure decreased E-cadherin protein levels normalized for β-actin, which was prevented by MAPC therapy. Cropped protein blots are shown for E-cadherin and β-actin (D). Full-length blots are shown in Supplementary Fig. 2. LPS exposure led to a biological relevant decrease in RAC in the RLL of prenatally assessed animals, which was resolved by MAPC treatment (E). In line, MAPC treatment increased fetal lung TTF-1+ AECs in the presence of prenatal inflammation in the RLL (F). Also in the RUL, MAPC treatment increased fetal lung TTF-1+ AEC2 in the presence of prenatal inflammation compared to controls and untreated animals (G). However, mRNA levels of the distal development driver SOX9 did not differ between groups of the prenatal cohort (H). Postnatally, no changes in RAC (I) and AEC2 (J) were measured. During postnatal assessment, MAPC administration following LPS exposure induced SOX9 mRNA expression in ventilated lambs (K). Data are presented as median with IQR. *OCLN* and *SOX9* mRNA expression and E-cadherin protein levels are displayed as relative fold changes over Sham-Saline-MV. Statistical significance was determined with a Kruskal-Wallis test followed by Dunn's multiple comparison and depicted as #p < 0.1, ∗p < 0.05, ∗∗p < 0.01. IQR = interquartile range, MAPC = multipotent adult progenitor cells, MV = mechanical ventilation, RAC = radial alveolar count, RLL = right lower lobe, RUL = right upper lobe.

The observed inflammatory response, with infiltrating leukocytes, can result in the disruption of the endothelial and epithelial integrity, resulting in the formation of edema. After LPS exposure and MV excess of fluid or remnants thereof were observed in both RUL and RLL compared to controls (RUL: *p* = 0.012 Sham-Saline-MV vs LPS-Saline-MV; Fig. 5A; RLL: *p* = 0.012 Sham-Saline-MV vs LPS-Saline-MV; Fig. 5B). MAPC treatment resulted in a biologically relevant reduction in the amount of accumulating fluid (RUL: *p* = 0.063 LPS-Saline-MV vs LPS-MAPC-MV; Fig. 5A; RLL: *p* = 0.075 LPS-Saline-MV vs LPS-MAPC-MV; Fig. 5B). Interestingly, fluid accumulation in LPS-exposed animals was paralleled by a significant decrease in mRNA levels of the tight junction molecule *OCLN* and a biologically relevant reduction in E–cadherin protein levels, a cell adhesion molecule, compared to non-exposed animals (*OCLN: p* = 0.002 Sham-Saline-MV vs LPS-Saline-MV; Fig. 5C; E-cadherin: *p* = 0.083 Sham-Saline-MV vs LPS-Saline-MV; Fig. 5D). MAPC treatment of ventilated LPS-exposed animals prevented this loss of cell junctions (*OCLN*: *p* = 0.015 LPS-Saline-MV vs LPS-MAPC-MV; Fig. 5C; E-cadherin: *p* = 0.023 LPS-Saline-MV vs LPS-MAPC-MV; Fig. 5D).

### 3.6 Alveolar development

Since LPS exposed lambs had a higher oxygen demand and alveolar simplification, is inherent to a reduced gas-exchange surface area in BPD patients [1], radial alveolar counts (RAC) were examined. The RAC of LPS-exposed animals was reduced in the RLL of the prenatal cohort compared to the control group (*p* = 0.05 Sham-Saline vs LPS-Saline; Fig. 5E). Following MAPC treatment a biologically relevant prevention of this reduction was observed (*p* = 0.06 LPS-Saline vs LPS-MAPC; Fig. 5E).

Next, we examined whether these changes were paralleled by effects on the number of TTF-1+ AEC2 in alveoli or on *SOX9* mRNA expression, which is a driver of distal lung development. Consistent with lower RAC in prenatally LPS-exposed lambs, the number of TTF-1+ cells in the RLL was decreased at birth (*p* = 0.017 Sham-Saline vs LPS-Saline; Fig. 5F). Interestingly, in the RUL, the number of TTF-1+ cells was increased in the LPS-MAPC groups compared to both the LPS- and control group (*p* = 0.035 Sham-Saline vs LPS-MAPC, *p* = 0.0003 LPS-Saline vs LPS-MAPC; Fig. 5G). In the RLL, MAPC also prevented the LPS-induced decrease in number of TTF-1+ cells (*p* = 0.042 LPS-Saline vs LPS-MAPC; Fig. 5F), but did not increase the number of TTF-1+ cells in comparison to control animals (Fig. 5F). Although the number of TTF-1+ cells was increased by MAPC treatment in lambs exposed prenatally to LPS, the mRNA expression of the distal developmental marker *SOX9* was not affected (Fig. 5H).

In ventilated animals, the RAC (Fig. 5I) and the number of TTF-1+ cells were unaffected by both prior LPS exposure and MAPC treatment (Fig. 5J). However, MAPC therapy following LPS exposure, resulted in an significant increase in *SOX9* mRNA levels compared to ventilated control animals (*p* = 0.004 Sham-Saline-MV vs LPS-MAPC-MV; Fig. 5K) and in a biologically relevant enhancement of *SOX9* mRNA expression compared to untreated animals (*p* = 0.099 LPS-Saline-MV vs LPS-MAPC-MV; Fig. 5K).

### 3.7 Principal component analysis

**Fig. 6:**
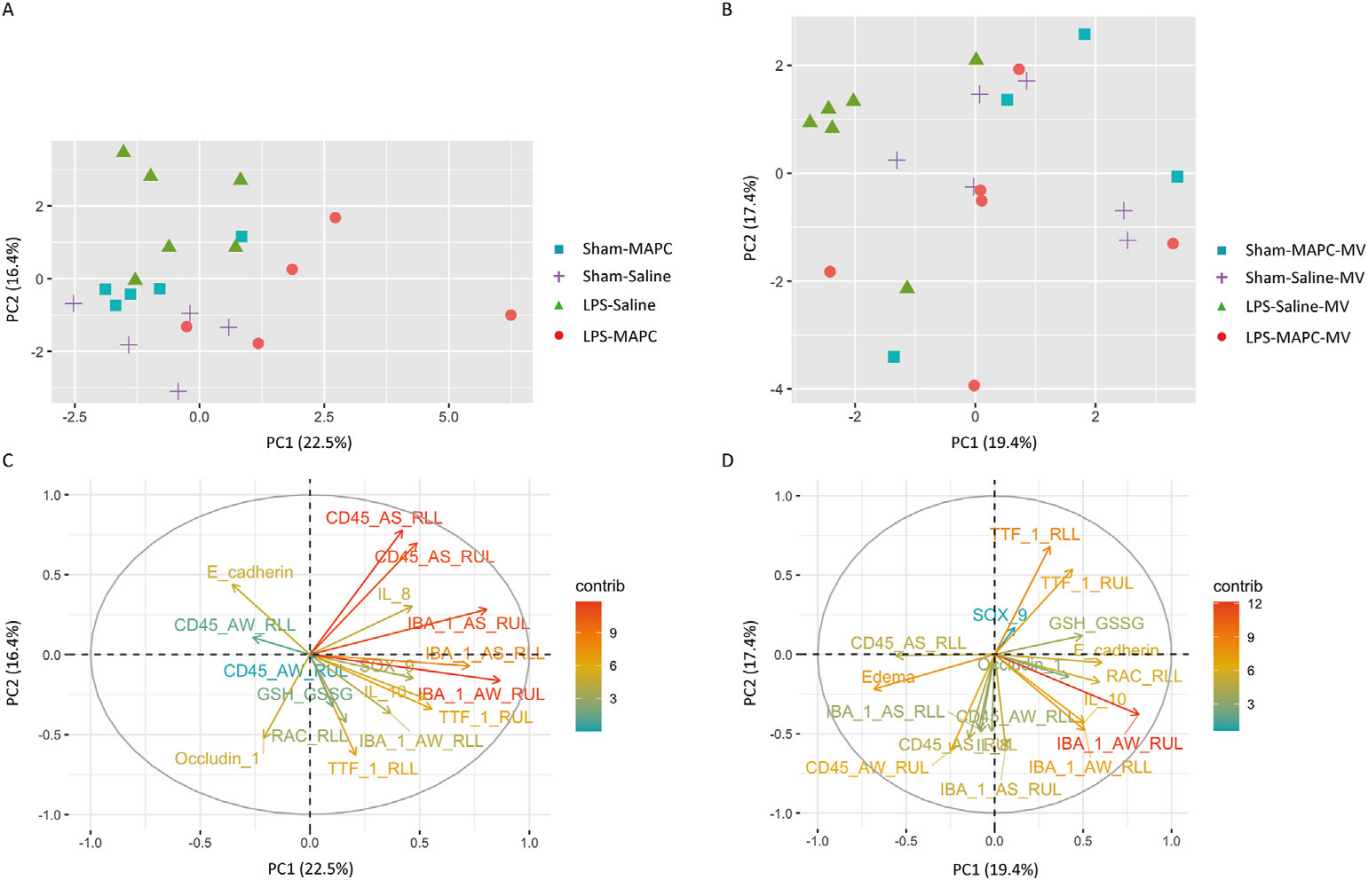
MAPC treatment shifts the immune reaction towards an anti-inflammatory response. Principal component analysis revealed that the second PC separated untreated LPS-exposed lambs from the other groups in the prenatal (A) and postnatal cohort (B). In the prenatal cohort, the LPS-MAPC group clearly clustered along a positive first PC (A), whereas postnatally, this segregation was less prominent (B). Pro-inflammatory vectors were driving the variance between Sham and LPS-exposed groups, which were inversely correlated with developmental, cell junctional and anti-inflammatory vectors contributing to clustering of the LPS-MAPC group in the prenatal study (C) and the postnatal study (D). AS = alveolar space, AW = alveolar wall, MV = mechanical ventilation, PC = principal component, RAC = radial alveolar count, RLL = right lower lobe, RUL = right upper lob.

Fig. 6A and B demonstrate a clear separation of experimental groups based on principal components. PCA visualization indicated that the number of CD45^+^ cells in AS of both the RUL and RLL and IBA-1 immunoreactivity in AS of the RUL were major contributors to the variance between Sham and LPS-exposed groups in the prenatal cohort. A positive correlation between IBA-1-positivity in the AW of both the RUL and RLL with the number of TTF-1+ cells in the RUL was found, driving the variance between the LPS-Saline and the LPS-MAPC group (Fig. 6C).

In ventilated animals, a positive correlation was found between pro-inflammatory markers (IL-8, AS CD45^+^ cells and AS IBA-1 immunoreactivity) and edema, which were the driving vectors for LPS-exposed animals to cluster along a positive first PC. These vectors were inversely correlated with TTF-1+ cells. Furthermore, IBA-1 immunoreactivity in the AW was the strongest contributor clustering unexposed and LPS-MAPC lambs towards a negative first PC. AW IBA-1 immunoreactivity was further correlated with RAC, *IL10*, GSH:GSSG ratio, E-cadherin and *OCLN* (Fig. 6D).

## 4 Discussion

In the current study, we investigated the pathophysiology underlying adverse preterm lung outcomes following perinatal inflammation and evaluated the efficacy of MAPC treatment in an ovine model of perinatal inflammatory stress comprising prenatal inflammation and postnatal MV around preterm birth.

First, prenatal pulmonary inflammation provoked by intra-amniotic LPS exposure contributed to impairments in lung development at birth. This sensitized the preterm lungs to a subsequent inflammatory hit (MV), resulting in increased oxygen demands and a higher A-a gradient. Impaired function was further aligned with persistent pulmonary inflammation and cell junctional loss, accompanied by pulmonary edema. Second, MAPC administration after initiation of systemic- and pulmonary inflammation, but before an additional postnatal hit, interrupted pulmonary inflammation and its negative sequelae. It even enhanced distal pulmonary development, demonstrating that MAPC therapy is not only capable of intercepting the cascade leading to adverse pulmonary outcomes, but can also compensate for developmental delays by stimulating maturation either directly or indirectly.

A quintessential hallmark of the multifactorial pathogenesis of BPD is an imbalance between pro- and anti-inflammatory mechanisms, favoring a pro-inflammatory environment [29,30]. Our model recapitulated this hallmark as shown by increased circulatory IL-6 and BALf IL-8 levels. Increased AS immune cell numbers, which were found both prenatally and postnatally, can be attributed in part to an elevation of macrophages/monocytes in AS of the RUL, indicative for newly recruited monocytes and suspected to be pro-inflammatory in nature [31]. Importantly, PCA results confirm that an enhanced pro-inflammatory immune response initiates the pathological cascade resulting in adverse pulmonary outcomes following perinatal insults.

In LPS-exposed MAPC-treated lambs, increased numbers of CD45^+^ cells, including macrophages, were present *in utero* in the AW. This indicates a MAPC-induced shift in the pulmonary immune response towards an anti-inflammatory reaction, as these cells have been associated with injury repair and support of regulatory T-cells [27,28]. In addition, resident alveolar macrophages can attach to the AW to communicate with the alveolar epithelium and attenuate lung inflammation [31,32]. Consistently, the immune modulatory effects of MAPC are further supported by enhanced mRNA expression of the anti-inflammatory *IL10* in the lungs in ventilated LPS-MAPC lambs. Importantly, PCA displayed that immune modulatory macrophages are important determinants of the effect of LPS and MAPC treatment, both *in utero* and during postnatal MV, indicating that the immunomodulatory properties of MAPC are an important driver for the observed beneficial effects.

Inflammation and oxidative stress are interrelated pathophysiological mechanisms. Importantly, the underdeveloped antioxidant system of preterm infants predisposes them to oxidative stress [33,34]. Our study indicates that MAPC increased GSH redox potential in BALf of LPS-exposed and non-exposed animals. This observation corresponds to previous findings of MSC that increase GSH levels in lung tissue [35,36] and could thereby prevent the development of pulmonary fibrosis in a bleomycin mouse model [37]. Moreover, PCA identified a positive correlation between the protective lung redox potential of MAPC and their immune modulatory effects.

Pulmonary inflammation, oxidative stress and mechanical injury predispose preterm infants to the disruption of epithelial and endothelial barriers and finally pulmonary edema and impaired gas exchange [38,39]. In line with this, LPS-exposed and ventilated animals exhibited more intra-alveolar exudate and congestion of AW. It is tempting to speculate that lung edema observed in LPS-exposed lambs is an underestimation of the actual edema that developed during MV, evidenced by the increased A-a gradients and oxygen requirements. Importantly, LPS-induced loss of cell junctions and pulmonary edema were obviated following MAPC therapy. Previous studies demonstrated that intra-tracheal or i.v. MSC treatment reduced the amount of excess water, which was initially caused by either LPS exposure or by postnatal ventilation [40,41]. Moreover, PCA suggests that both MAPC-induced modulation of the immune response and increase of GSH:GSSG levels in lungs prevented loss of epithelial/endothelial cell junctions and subsequent edema [33,42]. Collectively, the observed MAPC-driven reduction of pulmonary edema is considered involved in the improvement of pulmonary function. Assessment of multiple time points during the MV period could further elucidate pulmonary fluid accumulation and the concomitant endothelial- and epithelial variations in permeability.

The regenerative potential of MAPC is an essential factor contributing to improved postnatal pulmonary outcomes [43,44]. Namely, the improvement of lung function was preceded by enhanced acinar development after MAPC therapy, designated by increased RAC and TTF-1+ cells in LPS-exposed and MAPC-treated animals, indicating that an improved lung architecture in concert with the reduction of edema is responsible for the improvement of lung function. This concept is supported by earlier findings showing that MSC treatment accelerated pulmonary growth and regeneration in injured preterm lungs of mice and sheep [17,45]. For instance, a preterm lamb model of antenatal inflammation reported improved alveolar development by endorsing alveolar growth and differentiation after MSC treatment [17]. Postnatally, the improvements in pulmonary development continued during this study, as the expression of the distal lung progenitor marker *SOX9* was enhanced in the MAPC-treated LPS-exposed group. *SOX9* is expressed by epithelial progenitors that give rise to AE1C and AE2C [46,47]. The identified elevated *SOX9* expression, together with unchanged numbers of TTF-1+ cells, are indicative for an increased differentiation of the AE2C into AE1C, which could ultimately have contributed to a greater gas exchange surface in the alveoli, as seen upon MAPC therapy.

Interestingly, we detected significant regional distinctions within the lung for both developmental and inflammatory markers, endorsing the importance of examining different lung regions. The cranial part of the lungs is known to develop faster than the caudal part of the lungs in mammals [15]. These developmental stretches can also explain the observed differences between the upper and lower lobe. Moreover, the connection of the RUL bronchus to the trachea is above the carina in sheep, making the RUL more prone to atelectotrauma [15]. The arising perfusion-ventilation mismatch can be responsible for the observed regional variance within the lung, as oxygen concentrations are well known to modulate the immune response and stem cell function [33,48].

Despite the easier translation of research findings from large animals to the human situation, these large models also have limitations, including small group size and larger variations within groups. However, this preclinical ovine model allows for detailed itemization of multiple hits, including preterm birth, antenatal inflammation and postnatal MV, contributing to adverse pulmonary outcomes, whereas rodent models mostly focus on general lung structures following only one of these hits [49,50]. Additionally, the current model closely recapitulates the human situation, with comparable pulmonary development and MV. Here, we miss the investigation of temporal dynamics of the found pulmonary modifications in and beyond the 72 h of MV in the preterm lungs. The variety in pre- and postnatal effects indicates that different stimuli, particularly prenatal inflammation versus the combination with postnatal MV, can modulate the effects of MAPC in a time-dependent manner. Examining multiple time points during the ventilation period could possibly enlighten immune modulatory changes, developmental alterations and endothelial and epithelial variations, which we can only appraise at this point. Furthermore, it would also be of great importance to study a repetitive treatment regime, given the involvement of multiple pre- and postnatal inflammatory insults in the etiology of BPD.

## 5 Conclusion

In conclusion, we demonstrate that intra-amniotic LPS exposure initiates pulmonary inflammation, which continued postnatally and was inversely correlated to decreased alveolar development and cell junctional loss, predisposing the preterm lung to functional impairment. While oxygen therapy is required for the immature lung, it results in a subsequent postnatal hit, contributing to cell junctional loss accompanied by pulmonary edema. Administration of MAPC between prenatal and postnatal insults not only effectively prevented these pathological changes, but also induced maturation in the developing alveoli. Early application of MAPC therapy avoided inflammation-driven lung exacerbation and, hence, emerges as an effective treatment to reduce adverse lung outcomes following prematurity, including BPD.

## Funding

This work was financially supported by the 10.13039/501100015084Dutch Lung Foundation (Grant no. 6.1.16.088 to PGJN, NLR, and TGAMW). Multipotent adult progenitor cells were provided by Athersys Inc, Cleveland, Ohio. Athersys Inc. was not involved in the experimental design, (statistical) analysis, data presentation, or decision to publish. Poractant alfa Curosurf® was provided by Chiesi Farmaceutici S.p.A., Parma, Italy. Chiesi Pharmaceuticals was not involved in the experimental design, (statistical) analysis, data presentation, or decision to publish.

## Declaration of competing interest

The authors declare that they have no known competing financial interests or personal relationships that could have appeared to influence the work reported in this paper.

## Figures and Tables

**Supplementary Fig. 2:**
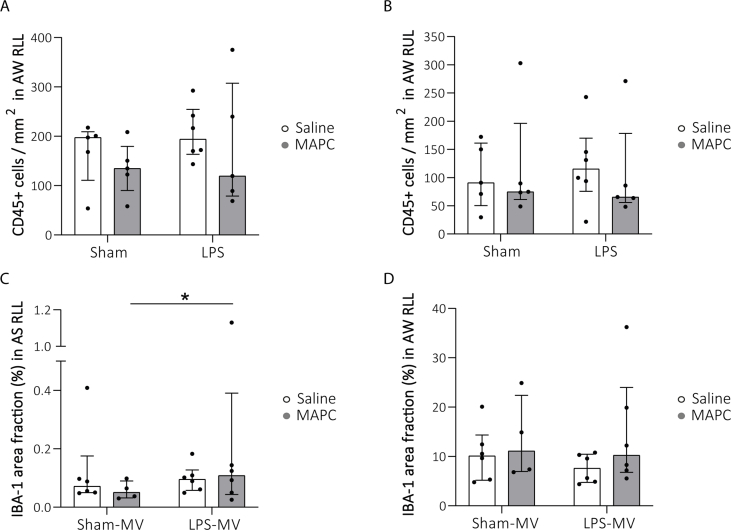
No changes in CD45^+^ immune cells were demonstrated in animals assessed directly at birth for the AW of the RLL (A) and the RUL (B). LPS exposure combined with postnatal MV, did not alter IBA-1immunoreactivity in both the AS (C) and the AW (D) of the RLL. Data are presented as median with IQR. Statistical significance was determined with a Kruskal-Wallis test followed by Dunn's multiple comparison and depicted as #*p* < 0.1, ∗*p* < 0.05, ∗∗*p* < 0.01. AS = alveolar space, AW = alveolar wall, IQR = interquartile range, MAPC = multipotent adult progenitor cells, MV = mechanical ventilation, RLL = right lower lobe, RUL = right upper.

**Supplementary Fig. 3:**
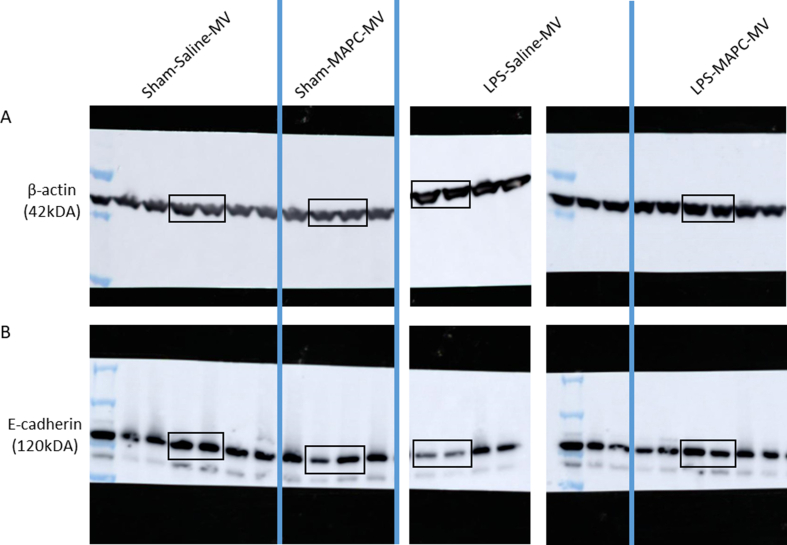
Western blot of E-cadherin and β-actin. Full length Western blot of β-actin at 42kDA (A) and of E-cadherin at 120kDA (B). Crop-positions are indicated within full-length blots with rectangles.

**Supplementary Fig. 4:**
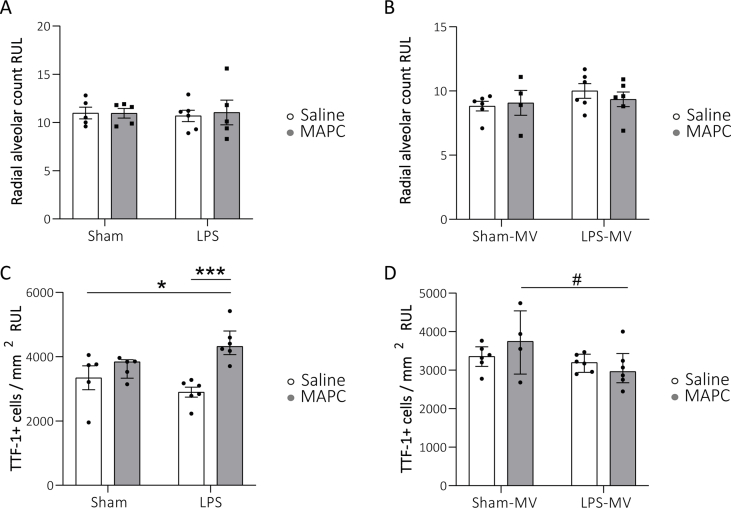
MAPC therapy following antenatal LPS exposure increases the number of AEC2. RAC of the RUL were not different between groups neither for the prenatal cohort (A) nor for the postnatal cohort (B). However, MAPC treatment increased fetal lung TTF-1+ AEC2 in the presence of prenatal inflammation in the RUL compared to controls and untreated animals (C). In MAPC-treated ventilated animals, the number of TTF-1+ AEC2 cells was biologically relevant decreased following intrauterine LPS exposure (D). Data are presented as median with IQR. Statistical significance was determined with an Kruskal-Wallis test followed by Dunn's multiple comparison and depicted as #*p* < 0.1, ∗*p* < 0.05, ∗∗∗*p* < 0.001. IQR = interquartile range, MAPC = multipotent adult progenitor cells, MV = mechanical ventilation, RAC = radial alveolar count, RUL = right upper lobe.
