# Supplementary material for: Multipotent adult progenitor cells prevent functional impairment and improve development in inflammation driven detriment of preterm ovine lungs
Source: Regen Ther. 2024 Mar 28;27:207–17. doi: 10.1016/j.reth.2024.03.014 (PMC10990734; doi:10.1016/j.reth.2024.03.014)
Supplement: Multimedia component 1 [file mmc1.docx]

**Supplementary Table 1. Primer-sequences for RT-qPCR.**

| Gene | Forward 5’ Primer-sequence | Reverse 5’ Primer-sequence |
| --- | --- | --- |
| *GAPDH* | GGAAGCTCACTGGCATGGC | CCTGCTTCACCACCTTCTTG |
| *ovRPS15* | CGAGATGGTGGGCAGCAT | GCTTGATTTCCACCTGGTTGA |
| *YWHAZ* | TGAACTCCCCTGAGAAAGCC | TCCGATGTCCACAATGTCAAGT |
| *IL10* | CATGGGCCTGACATCAAGGA | CGGAGGGTCTTCAGCTTCTC |
| *OCLN* | CCCGCCTGGCAAATGAC | GGATGACATGGCTGGGTTG |
| *SOX9* | CTCTGGAGACTGCTGAACGAG | GCCGTTCTTCACCGACTTCC |

*GAPDH = Glyceraldehyde 3-phosphate dehydrogenase, ovRSP15 = ovine ribosomal protein S15, YWHAZ = Human 14-3-3 protein zeta/delta, IL = interleukin, SOX = SRY-related HMG-box.*

**Supplementary Table 2. Western blot, ELISA & immunohistochemistry antibodies.**

| ELISA | | | |
| --- | --- | --- | --- |
| Coating antibody | **Standard curve** | **Detection antibody** | **Visualization antibody** |
| Mouse-anti-ovine  (MAB1004, Millipore Corporation, Darmstadt, Germany) | Recombinant IL-6  (6496, ImmunoChemistry Technologies, Bloomington, MN, USA) | Rabbit-anti-ovine IL-6  (AB1839, Millipore Corporation, Darmstadt, Germany) | Goat-anti-rabbit peroxidase-conjugated antibody  (111-035-045, Jackson ImmunoResearch, Cambridgeshire, UK) |
| Mouse-anti-ovine  (MAB1044, Millipore Corporation, Darmstadt, Germany) | Ovine IL-8  (6542, ImmunoChemistry Technologies, Bloomington, MN, USA) | Rabbit-anti-ovine IL-8  (AB1840, Millipore Corporation, Darmstadt, Germany) | Goat-anti-rabbit peroxidase-conjugated antibody  (111-035-045, Jackson ImmunoResearch, Cambridgeshire, UK) |
| Immunohistochemistry | | | |
| Primary antibodies | | **Secondary antibodies** | |
| Mouse-anti-CD45  (MCA2220GA, Bio-Rad, Veenendaal, the Netherlands) | | Biotin-labeled donkey-anti-mouse  (715-065-150, Jackson ImmunoResearch, Cambridgeshire, UK) | |
| Rabbit-anti-IBA-1  (019-19741, WAKO Pure Chemical Industries, Neuss, Germany) | | Biotin-labeled donkey-anti-rabbit  (711-065-152, Jackson ImmunoResearch, Cambridgeshire, UK) | |
| Mouse-anti-TTF-1  (6051116, Novocastra, Buffalo Grove, IL) | | EnVision FLEX /HRP  (Code K8002, DAKO Agilent Technologies, Abcoude, the Netherlands) | |
| Western blot | | | |
| Primary antibodies | | **Secondary antibodies** | |
| Rabbit-anti-E-cadherin (3195, Cell Signaling Technology Inc., Leiden, the Netherlands) | | Goat-anti-Rabbit IgG (BA10001.5, Vector Laboratories Inc., Newark, CA, USA) | |
| Mouse-anti β-actin (A5441, Sigma-Aldrich, Darmstadt, Germany) | | Horse-anti-Mouse IgG (BA2001.5, Vector Laboratories Inc., Newark, CA, USA) | |

*IL = interleukin, CD = cluster of differentiation, IBA-1 = ionized calcium-binding adapter molecule 1, TTF-1 = thyroid transcription factor 1.*

**Supplementary Table 3. Edema scoring criteria.**

| 0 | 1 | 2 | 3 |
| --- | --- | --- | --- |
| no fluids in the alveolar walls, thin alveolar membranes, rich in cell nuclei | beginning of pulmonary congestion by filling of hilar region, less dense nuclear presence | progression of fluid accumulation in interstitial walls, alveolar walls clearly thickened | severe edema, with alveolar flooding, distant cell nuclei, thick alveolar walls |
